# Stress Urinary Incontinence Among Young Nulliparous Female Athletes

**DOI:** 10.7759/cureus.17986

**Published:** 2021-09-15

**Authors:** Christine Joseph, Kosha Srivastava, Olive Ochuba, Sheila W Ruo, Tasnim Alkayyali, Jasmine K Sandhu, Ahsan Waqar, Ashish Jain, Sujan Poudel

**Affiliations:** 1 Urology and Obstetrics & Gynecology, California Institute of Behavioral Neurosciences & Psychology, Fairfield, USA; 2 Neurology, California Institute of Behavioral Neurosciences & Psychology, Fairfield, USA; 3 Internal Medicine, California Institute of Behavioral Neurosciences & Psychology, Fairfield, USA; 4 General Surgery Research, California Institute of Behavioral Neurosciences & Psychology, Fairfield, USA; 5 Pathology, California Institute of Behavioral Neurosciences & Psychology, Fairfield, USA; 6 Obstetrics & Gynecology, California Institute of Behavioral Neurosciences & Psychology, Fairfield, USA; 7 Family Medicine, California Institute of Behavioral Neurosciences & Psychology, Fairfield, USA; 8 Psychiatry and Behavioral Sciences, California Institute of Behavioral Neurosciences & Psychology, Fairfield, USA

**Keywords:** female athlete, stress urinary incontinence, pelvic floor muscles, nulliparous, exercise, prevalence, association

## Abstract

Urinary incontinence (UI) is described as unintentional voiding of urine that is usually seen in post-partum and post-menopausal women due to the weakening of pelvic floor muscles (PFM). Recent studies have shown an increase in the prevalence of stress urinary incontinence (SUI) among young nulliparous female athletes. The association between UI and high-impact physical activity is due to increased intra-abdominal pressure during high-impact sports exceeding intra-urethral pressure. Usually, the levator ani muscle (LAM) helps in urethral closure. However, weakening or injury of LAM can reduce the pelvic support and cause UI in young female athletes. This study aims to assess the prevalence of SUI among young nulliparous athletes and also explore the association between SUI and athletic sports in young females. We searched PubMed and Google Scholar as databases to find specific articles about the topic. After the inclusion and exclusion criteria were applied, 52 articles were selected for this review. It is found that there is an increased UI prevalence, mainly SUI, among young nulliparous female athletes, especially in volleyball players and long-distance runners. Nulliparous athletes involved in high-impact exercises were found to have an increased cross-sectional area of LAM and puborectalis muscle width. SUI is usually under-reported and underdiagnosed due to lack of knowledge and unawareness, which can negatively affect the personal and social life of young females. PFM training is considered the first line of therapy among nulliparous athletes. However, it is unclear whether the high-impact effects of sports cause UI through PFM fatigue or PFM damage. More research is needed to better understand this effect.

## Introduction and background

Urinary incontinence (UI) is described as an unintentional voiding of urine, which is most commonly seen in post-partum and post-menopausal women due to the weakening of the pelvic floor muscles (PFM) [1]. It is two to four times more frequent in women than in men, with a prevalence of about 20%-40% [2]. There are several types of UI, of which stress urinary incontinence (SUI) is the most common [3]. SUI occurs in situations of increased abdominal pressure such as coughing, lifting weights, exertion, sneezing, jumping, and squatting. Although it is more commonly seen in multiparous women, recent studies have shown increased SUI prevalence among young nulliparous female athletes [1]. A systematic review and meta-analysis conducted in 2020 found a prevalence rate of UI among female athletes to be about 25.9% in different sports, with SUI being the most common. Among that, volleyball players were found to have the highest UI prevalence rate of 75.6% [3]. Although physical activity is known to strengthen PFM, overstretching and overstraining of PFM can cause the opposite effect [4]. One of the recent studies has proposed the association between UI and high-impact physical activity due to an increased intra-abdominal pressure that builds up during high-impact sports to a level that exceeds the intra-urethral pressure [5]. Similarly, an imbalance in the abdominal force can lead to a change in the physiological urethrovesical angle leading to UI, primarily SUI in high-impact athletes [6]. However, most athletes experience leakage during training and not during a competition (95.2% vs 51.2%, respectively), probably due to higher catecholamine levels affecting the urethral alpha receptors, maintaining urethral closure during competitions [7].

The PFM primarily supports the abdominal and pelvic structures, including maintaining urinary and fecal continence [8]. Levator ani muscle (LAM), made of iliococcygeus, puborectalis, and pubococcygeus, is the primary muscle that protects the pelvis [8]. Usually, during increased intra-abdominal pressure, the LAM helps urethral closure by allowing it to compress on it firmly [9]. However, studies have found that LAM injury can reduce urethral support, mainly associated with SUI [9]. Few results have proved an increase in LAM cross-sectional area and puborectalis muscle width in nulliparous females taking part in strenuous activity using magnetic resonance imaging (MRI) [10-12]. It is perceived that increased intra-abdominal pressure results in stress-induced hypertrophy [10-12]. Similarly, 24 nulliparous athletes were found to have a larger levator hiatus area and greater bladder descent during Valsalva using three-dimensional/four-dimensional translabial ultrasound [13]. Several studies managed to search for pelvic floor dysfunction during high-impact exercise using methods like perineometer and vaginal squeeze pressure that were used to determine the maximum voluntary contraction (MVC), which is the standard gold method to assess the pelvic muscle fatigue described as "a person's effort to engage as many muscle fibers as possible to exert force" [14]. Ree et al. stated that nulliparous women involved in the strenuous exercise who had SUI were found to have lower maximum voluntary vaginal contraction pressure that stipulates PFM exhaustion [15]. An observational study in 2013 concluded a decrease in perineal pressure among female athletes compared to non-athletes, which correlates with increased UI symptoms [16]. The American Urogynecologic Society's website recommends that women avoided heavy lifting and repeated high-impact activity that causes an increase in abdominal pressure to prevent pelvic floor dysfunction [17]. Low-impact exercises, such as walking, are associated with lower SUI prevalence [17].

UI among nulliparous athletes is often underdiagnosed and under-reported [1]. Despite the high prevalence of UI among athletes, women do not usually address the problem or go to a specialist because of embarrassment and lack of knowledge about acquiring UI [1]. Approximately 23% of incontinent women either stop or reduce the amount of exercising due to UI, and 60% of women with severe UI are likely to be inactive [18]. Although physical activity has several health benefits, female athletes may be hesitant to remain active. Lack of proper guidance may cause women to stop exercising because of urinary incontinence or fear that such activity will promote pelvic floor disorders [19]. A few standard methods are available for treating UI, such as physiotherapy, biofeedback, pharmacological, and in severe cases, surgery [20].

We searched PubMed and Google Scholar databases to find relevant articles. Inclusion and exclusion criteria were applied. The inclusion criteria included articles from 2010 to 2021 and had several types of clinical trials, observational studies, review articles, systematic review, meta-analysis, and randomized control trials (RCT). We searched only for human studies with ages between 18 and 44 years. We excluded all the animal studies, studies written in a non-English language, studies with patients less than 18 years, and articles published before 2010. After applying the inclusion and exclusion criteria, 227 articles were found. Screening of titles was done, which narrowed the search to 80 relevant articles. Duplicates were screened among these articles, and 57 studies remained. A total of 52 articles were used after a thorough screening of abstracts and full texts.

This review article aims to assess the prevalence of SUI among young nulliparous female athletes in recent years and to explore how SUI may be associated with sports activities in young females. We also reviewed the impact that SUI may have on the well-being of female athletes and the currently available treatments.

## Review

Prevalence of SUI in different types of sports

Athletes involved in high-impact exercises cause increased pressure in the pelvic muscles leading to an increased UI prevalence. A meta-analysis done by Sorrigueta-Hernández et al. in 2020 formed three study groups according to the impact of sports on PFM [6]. The prevalence of UI was 36.4%, 50%, and 64% in female athletes playing low-impact, moderate-impact, and high-impact groups, respectively [6]. Yang et al. in 2019 conducted a cross-sectional study confirming the high prevalence of athletes involved in cross-fit exercises, including double under (47.7%), jumping rope (41.3%), and box jumps (28.4%), compared to aerobic athletes [21]. In 2018, an observational study was conducted by Hagovska et al. that demonstrated UI in 33 (6.14%) sportswomen compared to 11 (2.04%) in non-sportswomen [22]. A cross-sectional study (2018) had further concluded an increase in SUI prevalence among volleyball players as compared to other sports [23]. Hagovska et al. (2017) performed a cross-sectional study consisting of 503 sportswomen and reported a prevalence of 72 (14.3%) athletes using several questionnaires, among which SUI was the most common (13.52%) [24]. An online survey was done among 311 triathletes in 2016 by Yi et al. showing an SUI prevalence rate of 37.4% among female athletes [25]. A total of 22 trampolinists had participated in a cohort study (2015) confirming urinary leakage of about 72.7% using a questionnaire [11]. Borin et al. (2013) divided 40 women into four categories: 10 (volleyball players), 10 (basketball players), 10 (handball players), and 10 non-athletes [16]. Intracavity pressure was carried out using a perineometer and perineal pressure for each group proved that perineal pressure was reduced in athletes compared to non-athletes [16]. A lower perineal pressure corresponds to an increased chance of developing SUI [16]. A prospective study (2012) divided 488 nulliparous women into those who attended gym (study group) and those who did not (control group) [26]. Using questionnaires, results revealed a mean of 1.68 in the study group compared to a mean of 1.02 in the control group [26]. Dos Santos et al. performed a cross-sectional study (2019) during one hour of sports training using a modified pad test to quantify the urine loss in high-impact exercise [10]. Results showed that 43.7% of athletes had urine leakage during the training pad test with a mean loss of 1.57 ± 0.4 g of urine [10]. However, 24% of athletes who did not complain of UI also had a positive pad test result [10].

Table 1 below summarizes the UI prevalence among different types of sports, mainly showing an increased prevalence among volleyball players, trampolinists, athletes participating in cross-fit exercises, and those involved in high-impact training.

**Table 1:** Prevalence of SUI in recent years. SUI, stress urinary incontinence; IPAQ, International Physical Activity Questionnaire; ICIQ-SF, International Consultation on Incontinence Questionnaire - Short Form; ICIQ-UI-SF, International Consultation on Incontinence Questionnaire Urinary Incontinence - Short Form; OAB-Q, Overactive Bladder Questionnaire; I-QOL, Urinary Incontinence Quality Of Life scale; FATQ, Female Athlete Triad Questionnaire; PGQ, Pelvic Girdle Questionnaire; EPIQ, Epidemiology of Prolapse and Incontinence Questionnaire; NA, not applicable.

AUTHOR AND YEAR OF PUBLICATION	SAMPLE	STUDY DESIGN	ASSESSMENT	AGE	OUTCOME
Sorrigueta-Hernández et al. (2020) [6]	G1 (low-impact), G2 (moderate-impact sports), and G3 (high-impact sports)	Meta-analysis	NA	22.6 years	Prevalence of UI in G1 (36.4%), G2 (50%), and G3 (64%).
Yang et al. (2019) [21]	105 women (cross-fit exercise) and 44 women (aerobic)	Cross-sectional	NA	36 years (cross-fit group), 29 years (aerobic group)	50 women had SUI during training in cross-fit exercise that includes double under (47.7%), jumping rope (41.3%), and box jumps (28.4%). No SUI was observed in women performing aerobics while training.
Hagovska et al. (2018) [22]	270 (sportswomen) and 287 (non-sportswomen)	Observational study	IPAQ, ICIQ-UI-SF, OAB-Q, and I-QOL	20.9 ± 2.8 years	Urinary leakage observed in sportswomen was 33 (6.14%) as compared to non-sportswomen (11, 2.04%). SUI was found prevalent among women who practiced high-impact training.
Hagovska et al. (2018) [23]	278 sportswomen	Cross-sectional	IPAQ, ICIQ-UI-SF, OAB-Q, and I-QOL	NA	SUI in athletes was 23.8%, especially in volleyball players of about 19.6%.
Hagovska et al. (2017) [24]	503 sportswomen	Cross-sectional	IPAQ, ICIQ-SF, OAB-Q, and I-QOL	21.1 ± 3.6 years	Urinary leakage was seen in 72 sportswomen (14.3%) and SUI was seen in 62 women (13.52%).
Yi et al. (2016) [25]	311 triathletes	Online survey	FATQ, PGQ, and EPIQ	NA	The prevalence of SUI in triathletes was 37.4%.
Da Roza et al. (2015) [11]	22 trampolinists	Cross-sectional cohort study	ICIQ-UI-SF	NA	Urine leakage was confirmed in 72.7% of participants during the practice.
Borin et al. (2013) [16]	40 women were divided into four categories: 10 (basketball players), 10 (volleyball players), 10 (handball players), and 10 (non-athletes)	Observational study	Questionnaire	18-30 years	Perineal pressure was measured using a perineometer and showed for non-athletes (6.73 ± 1.91 mm Hg), handball players (5.55 ± 1.43 mm Hg), volleyball players (4.36 ± 1.43 mm Hg), and basketball players (3.65 ± 1.35 mm Hg). The lower the perineal pressure, the higher the chance to acquire UI and pelvic floor dysfunction.
Fozzatti et al. (2012) [26]	488 nulliparous women were divided into the study group (those that attended gym) and the comparative group (those that did not)	Prospective comparative study	ICIQ-SF	NA	The average result of SUI in the study group was 1.68 (+3.46) and the comparative group was 1.02 (+2.69).

Association of SUI and nulliparous athletes

Urinary continence is generally formed by the balance between intravesical and intraurethral pressure [27]. Intravesical pressure is determined by the intraabdominal pressure, whereas intraurethral pressure is influenced by the sphincter and "hammock" muscle (levator ani, anterior vagina wall) [27]. In case of increased intra-abdominal pressure, the mechanism modulating urethral closure involves shortening the sub-urethral part of the vaginal wall and levator ani, with sacrouterine ligaments pulling in the posterior direction [27]. Dysregulation of this delicate balance can lead to UI in young women [27]. SUI is more prevalent in high-impact exercises as compared to low-impact ones. High-impact exercises are described as exercises that engage in lower extremity weight-bearing and activities that involve both feet held off the ground simultaneously with a rapid increase in intra-abdominal pressure [26]. The "hammock hypothesis" states that an increase in intra-abdominal pressure causes a stretch to the pelvic ligaments and floor muscles, leading to muscle fatigue and permanent tissue damage [23,28]. Moreover, muscle fatigue can cause an imbalance between the downward force from the abdomen and upward pressure from PFM, leading to SUI [21]. The study also suggests that SUI related to muscular fatigue causes leakage more frequently late during the day and during high-impact exercises [21]. There are two conflicting theories suggestive of PFM in athletes. The first theory states that PFM is stronger in athletes than non-athletes due to repeated use through training, and the second theory states that repeated intra-abdominal pressure on the pelvic floor can weaken PFM, causing stretching of the fascia and muscles [29]. A recent study found stronger pelvic muscles in athletes with UI than without UI [20]. It may be due to solid pelvic muscles that fail to relax fully and increase stress leading to increased urinary frequency and eventually UI [30]. In the past, jumping was thought to have the highest prevalence for UI since it is a significant factor for PFM weakening [1]. However, it has been recently shown to have the lowest UI prevalence [1]. Subsequently, long-distance running is more likely to cause high UI prevalence due to the prolonged duration of force on PFM [31]. UI affects physical health and may negatively influence the adolescent's psychological well-being [32,33]. For example, a cross-sectional study conducted by Santos et al.(2018) reported a high prevalence of UI (48%) among 50 nulliparous athletes above 18 years of age [34]. Furthermore, about 44% of these athletes showed sexual dysfunction, associated with a reduced sexual desire in UI [34].

Management of SUI

The primary aim of UI treatment is to regain continence. Therefore, management begins with conservative measures and changes in lifestyle such as voiding before workouts, avoiding fluids just before physical activity, wearing dark-shaded garments, followed by physiotherapy of the PFM and pharmaceutical treatments. Surgery is reserved for refractory or severe cases [27].

Non-Pharmacological Methods

PFM strength training is proven to treat UI among women and is recommended as a primary therapy [35]. The exercises done to strengthen the PFM include phasic and tonic contraction. Phasic contraction provides immediate support to the urethra, whereas tonic contraction stabilizes the urethra [27]. An observational study conducted in 2020 demonstrated SUI improvement in athletes that underwent pelvic muscle training compared to those that did not [36]. Furthermore, a pilot study done in 2012 showed an improvement in the vaginal resting pressure and MVC in athletic students who completed an eight-week program of PFM training [37]. Similarly, an RCT was conducted in 2020, dividing women with SUI that never had physiotherapy done before into those who underwent PFM training and those who had received the abdominal hypopressive technique [38]. Using questionnaire responses, researchers had shown that both the groups had an improvement in SUI; however, PFM training was found to be superior over abdominal hypopressive technique [38]. The training consists typically of regular contraction and relaxation of the pelvic muscles, also known as Kegel exercises. They are well known to improve muscle tension and blood supply [8]. However, a study conducted in 2019 revealed that a group of gymnasts were not aware of PFM training [35]. Most young athletes were not able to contract the PFM properly at their first attempt [35]. With this in mind, more studies are needed to assess the need for PFM training to be included in general strength training programs [35]. Suprapubic ultrasound is a definitive and non-invasive method to evaluate and teach PFM contractions [39]. An experimental study (2014) for the PFM rehabilitation program (PFMRP) was conducted on volleyball players by randomly assigning them into two groups: experimental group (EG) and control group (CG) [40]. The EG received the PFMRP that consists of awareness and identification of PFM as well as timed contractions, whereas the CG only received the pamphlet [40]. At the end of three months, by using questionnaires, it was found that urine leakage was reduced in 45.5% of athletes in EG compared to 4.9% in CG, and a significant improvement in UI symptoms was found [40]. Another method is biofeedback (BF), which is based on the self-control of physiological activities taking part in our body that were previously uncontrolled [8]. Therefore, self-monitoring can be included in BF therapy, thereby allowing for greater motivation levels and an awareness of the body's physiological state that may have been previously unknown [8]. BF is recently being used in SUI treatment, as this method increases the strength of PFM that was previously weakened. In this case, pelvic muscles are supported by the fascia in the pelvis [8]. In addition, BF also helps acquire information on the intensity and duration of PFM contractions [41]. An RCT in 2015 by Ong et al. divided 40 patients with SUI into a control group (PFM exercise alone) and a study group (using biofeedback Vibrance Kegel Device [VKD] with PFM exercise) [42]. After 16 weeks of training, questionnaire responses showed that the PFM strength was better in patients that used VKD (*P* = 0.001) [42]. Electromyography (EMG) is added to the BF method that records the electrical activities of the muscles and nerves [8]. Vaginal and surface electrodes are possible in this method that is placed in the lower abdomen and the perineum [8]. The PFM tension is carried out by these electrodes, which records them and then convey information regarding the contractions to the apparatus the patient is connected on [8]. The patient then monitors the quality of the contractions and performs Kegel exercises within the limits of physiological tension [8]. When the patient performs the exercises in the right way, visual and auditory stimuli get activated [8]. In an observational study done in 2018, an eight-week PFM training program with EMG was performed in women with SUI and showed a remarkable improvement in the voluntary contraction of pelvic muscles of women in different postures like sitting, standing, and in the supine position [43].

PFM training is considered the first line of treatment among nulliparous athletes and is proven to be highly effective. Thus, athletes should be educated on being able to contract PFM in the right way. BF, on the other hand, has gained its popularity only in recent years and is being used by professionals as a non-pharmacological therapy.

Pharmacological and Surgical Therapy

**Figure 1:**
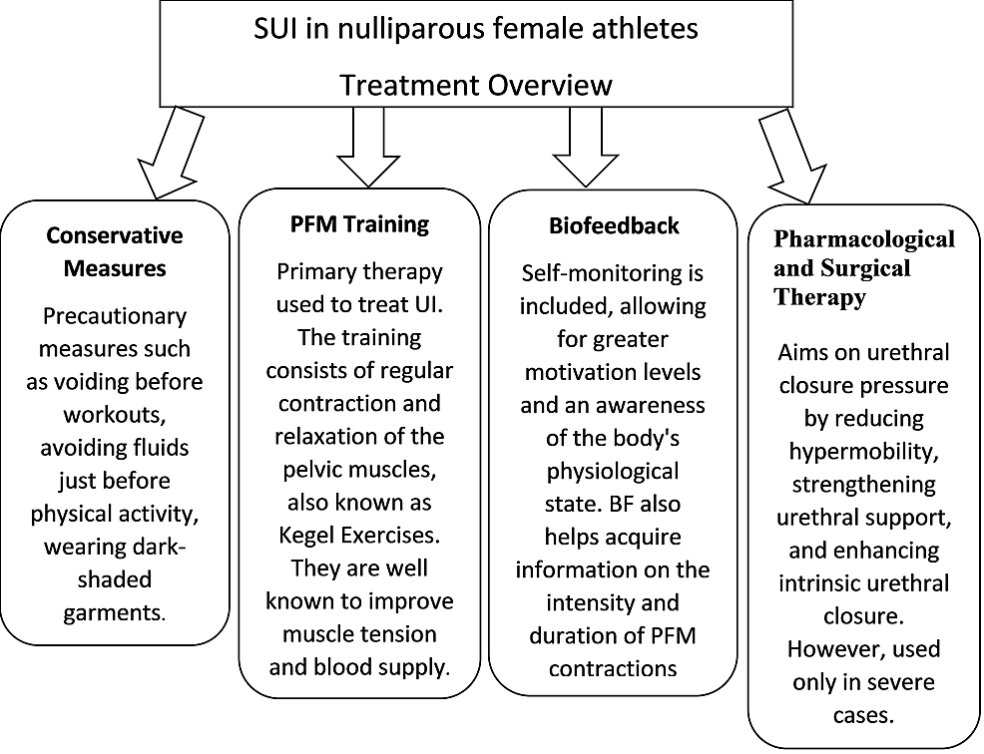
Management overview of SUI in female athletes. PFM, pelvic floor muscles; SUI, stress urinary incontinence; BF, biofeedback; UI, urinary incontinence.

SUI management focuses mainly on the urethral closure pressure by reducing hypermobility, strengthening urethral support, and enhancing intrinsic urethral closure [44]. Medications such as alpha-adrenoreceptor agonists (ephedrine, norepinephrine, and phenylpropanolamine), imipramine, and duloxetine are used [27]. Occasionally, estrogen is used in situations of hypoestrogenism [27]. The use of exogenous hormones may positively influence pelvic floor function by improving muscle quality [45]. However, young athletes do not use pharmacological management as they experience SUI only during high-impact activities and not during daily activities [27]. Although mid-urethral slings are a standard method for UI treatment, it is not practiced in young athletes, especially if the leakage occurs only during sports [27]. Pessaries and vaginal tampons are considered the only non-invasive methods used by athletes that most commonly offer proper urethral support during high-impact activity [27]. Figure 1 below shows an overview of the management of SUI among female athletes.

Benefits of physical activity

Physical activity has several benefits for the body, and women should not be discouraged from remaining active. Adding exercise as a part of lifestyle helps prevent non-communicable diseases such as diabetes mellitus, hypertension, hypercholesterolemia, and obesity [46]. An RCT done by Said et al. (2017) randomly divided 32 obese women into high-impact aerobics groups and low-impact aerobics combined with a strength training program group [47]. At the end of 24 weeks, there was a significant decrease in the total cholesterol and triglyceride levels, along with an improvement in the body composition and physical fitness in both groups [47]. However, weight loss and body fat reduction were observed in the high-impact aerobics group [47]. Obesity is known to cause SUI, and persistent exercise can lead to weight loss, thereby preventing UI progression [48]. Therefore, PFM training should be added to training classes and fitness clubs for women [49]. Poor cardiovascular well-being can be associated with poor bladder health. Reduced sensory stimuli carried by the autonomic nervous system and reduced blood flow through narrowed peripheral vasculature could decrease the functions of the bladder and PFM [50]. It is highly advised that fitness professionals educate women to perform simple modifications to supine exercises to strengthen their PFM [51]. Once women master the supine exercises, they should be taught to train the PFM in different postures, including standing to stabilize the surrounding pelvic muscles and hip girdles [51]. Furthermore, when women have learned to contract the PFM correctly, they consciously become aware of tightening the PFM during daily activities [51]. A case-control study was done in 2010 with 331 former athletes and 640 controls to investigate whether former athletes were prone to experience UI later in their life [52]. The results showed similar SUI prevalence rates among the former athletes and the non-athletic control group [52]. Therefore, it was concluded that high-impact exercises at an early age are not a risk factor for UI in later life [52], and young athletes should continue to practice sports with an emphasis on performing exercises that strengthen their PFM.

Limitations

The limitation of this paper is that it has only 11 years of data available. Thus most of the studies suggesting the risk factors and management could not be accessed. It also focuses only on SUI and does not highlight the prevalence of other UI types in young nulliparous women. Very few data were available exhibiting the treatment of UI among nulliparous athletes. Although we are well aware of the risk factors of UI in the general female population, such as pregnancy, vaginal delivery, obesity, and pelvic surgeries, the studies regarding risk factors for UI in young nulliparous women are preliminary.

## Conclusions

Our review article has shown that there has been an increased UI prevalence, predominantly SUI, among young nulliparous female athletes. It is more commonly seen in volleyball players and in long-distance runners. SUI occurs due to a repeated increase in intra-abdominal pressure leading to a weakened PFM. An injury to the LAM reduces urethral support, predisposing to UI. Nulliparous athletes involved in high-impact exercises were found to have an increased cross-sectional area of LAM and puborectalis muscle width. Greater awareness is needed to help combat psychosocial distress and speed up access to diagnosis and treatment. It is managed conservatively with few preventive strategies and PFM training. Pharmacological and surgical therapies are considered only in severe cases. Young athletes should be taught how to perform exercises in the right way. In the future, physicians, mainly those involved in women's healthcare, should be encouraged to educate patients, especially young athletes, about UI. Although there are two theories regarding PFM in young athletes, there has been no evidence supporting either of them. It is unclear whether the high-impact exercises cause SUI through muscle fatigue or muscle damage. More research in the coming years is needed to understand this observation better.
